# The impact of regular physical exercise on mobile phone addiction tendency among Chinese college students: the mediating role of trait boredom and the moderating role of self-control

**DOI:** 10.3389/fpsyg.2025.1671836

**Published:** 2025-10-20

**Authors:** Jiatian Qi, Xiaoyou Zhou, Jingjing Li, Bing Liu

**Affiliations:** ^1^Physical Education College, Shanghai University, Shanghai, China; ^2^School of Economics, Shanghai University, Shanghai, China

**Keywords:** regular physical exercise, mobile phone addiction tendency, trait boredom, self-control, college students

## Abstract

**Introduction:**

This study explores the relationship between regular physical exercise and mobile phone addiction tendency among college students, with a focus on the mediating role of trait boredom and the moderating role of self-control.

**Methods:**

A total of 560 students from three universities in Shanghai were surveyed using the Revised Physical Activity Rating Scale, the Exercise Stage Scale, the Trait Boredom Scale, the Brief Self-control Scale, and the College Students’ Mobile Phone Addiction Tendency Scale.

**Results:**

(1) regular physical exercise is significantly negatively correlated with mobile phone addiction tendency, suggesting that students who engage in more regular physical exercise exhibit lower levels of mobile phone addiction tendency; (2) trait boredom mediates the relationship between regular physical exercise and mobile phone addiction tendency; (3) self-control moderates the effect of trait boredom on mobile phone addiction tendency, such that higher levels of self-control weaken the positive association between trait boredom and mobile phone addiction tendency, whereas lower levels of self-control strengthen this association; (4) the moderating effect of self-control on the direct relationship between regular physical exercise and mobile phone addiction tendency is not significant.

**Discussion:**

This study reveals the underlying mechanisms through which regular physical exercise affects mobile phone addiction tendency and provides theoretical and practical implications for the design of intervention strategies and the cultivation of self-control among college students.

## 1 Introduction

According to the *Statistical Report on Internet Development in China* (Center, 2025), the number of internet users in China had reached 1.123 billion by December 2025, with 79.7% accessing the internet via mobile phones. Among these users, college students represent one of the most active demographic groups. Data show that 20.59% of college students spend more than 6 h per day immersed in digital environments such as instant messaging, short videos, and online games (Research, 2021), which significantly affects their mental health and daily functioning. Scholars have defined problematic or compulsive mobile phone use, which adversely impacts psychological, behavioral, and social functioning, as mobile phone dependence, problematic smartphone use, or mobile phone addiction (Liu et al., 2017). It is characterized as an impulsive, habitual behavioral response (Van Deursen et al., 2015). Studies report that the prevalence of internet addiction among Chinese college students is approximately 11.76%, with up to 58.33% at risk of developing addictive tendency (Xie et al., 2016). Mobile phone addiction has been identified as a key factor affecting students’ physical and mental well-being, associated with reduced sleep quality, increased risks of depression and anxiety, impaired interpersonal relationships, and diminished subjective well-being (Chen and Leung, 2016; Hou et al., 2021; Ze et al., 2022). Furthermore, mobile phone addiction among college students may lead to broader societal concerns, including social withdrawal, strained parent–child relationships, and declining marriage and birth rates (Huajian et al., 2020). Therefore, it is imperative to explore the underlying causes and mechanisms of mobile phone addiction among college students.

Current research has mainly examined the antecedents of mobile phone addiction from cognitive, emotional, family, and personality perspectives (Casale et al., 2021; Kim et al., 2018; Park and Lee, 2011). Many studies emphasize the role of negative emotion such as depression, anxiety, loneliness, and boredom in predicting mobile phone addiction tendency (Chen and Leung, 2016; Hou et al., 2021). The accessibility of mobile phones and their use in intermittent scenarios increase the likelihood of emotional regulation through mobile phone use, particularly when individuals experience boredom (Tong et al., 2019). In psychology, boredom is typically classified into state boredom and trait boredom. While state boredom refers to a temporary emotional experience in specific situations, trait boredom is considered a relatively stable personality disposition (Farmer and Sundberg, 1986). Individuals with high levels of trait boredom are more prone to negative emotions such as depression and anxiety, and studies have shown that trait boredom is a significant predictor of mobile phone addiction tendency among college students (Tong et al., 2019).

In response to mobile phone addiction among college students, increasing attention has been paid to the role of regular physical exercise. Studies have shown that engaging in regular physical exercise can effectively reduce mobile phone addiction tendency (Machado et al., 2022), alleviate negative emotion such as depression, anxiety, and boredom (Wang Y. et al., 2025; Wang Z. et al., 2025), and improve adverse personality traits like trait boredom, trait anxiety, and irritability, along with various forms of addictive behavior (Dong et al., 2022; Lu et al., 2022). Self-control has also been identified as a critical psychological factor closely linked to addiction. Numerous studies have confirmed that self-control is negatively associated with mobile phone addiction (Lin et al., 2025) and serves as a key protective factor in its prevention (Zhang et al., 2019).

While existing research has broadly affirmed the health benefits of regular physical exercise, such as reducing the risks of cardiovascular disease and obesity, and mitigating emotional distress, few studies have addressed the psychological mechanisms underlying the relationship between regular physical exercise and mobile phone addiction tendency. Trait boredom and self-control are two psychological variables that play an essential role in this process. Therefore, the present study aims to investigate the mechanisms through which regular physical exercise influences mobile phone addiction tendency among college students, with particular focus on the mediating role of trait boredom and the moderating role of self-control. The goal is to inform strategies to reduce problematic mobile phone use and promote healthier behavioral patterns in college students.

## 2 Literature review

### 2.1 Regular physical exercise and mobile phone addiction tendency

Regular physical exercise is widely recognized as a key factor influencing individuals’ physical fitness, psychological well-being, cognitive functioning, and behavioral regulation (Cho and Kim, 2020; White et al., 2024). Over the past decade, increasing attention has been paid to its potential role in mitigating various forms of addictive behaviors, including substance use, problematic internet use, and mobile phone addiction. Empirical evidence shows that structured exercise interventions can reduce cravings among individuals with substance use disorders (Suh et al., 2002), while higher levels of physical exercise are associated with lower levels of problematic internet use and excessive social networking (Koçak, 2019; Xiao, 2022). These protective effects have been attributed to improvements in self-regulation, cognitive flexibility, and emotional stability (Gellert et al., 2012; Li et al., 2023; Ye et al., 2022). Moreover, regular physical exercise can alleviate psychological stress and negative affect, thereby reducing the likelihood of maladaptive behaviors such as excessive smartphone use (Li and Dong, 2022; Liang and Jia, 2022).

In the Chinese higher education context, a growing body of research mirrors and extends these findings. Recent studies consistently report a negative association between physical exercise and mobile phone addiction among college students, suggesting that the mechanisms observed internationally are also applicable in China. Specifically, higher levels of regular physical exercise are linked to lower smartphone dependence through enhanced self-control, reduced perceived stress, and improved sleep quality (Wang et al., 2024; Ye et al., 2025). Physical exercise has also been shown to promote learning engagement and psychological well-being via increased academic self-efficacy and positive emotions, which indirectly contribute to reduced mobile phone addiction tendencies (Meng et al., 2025; Zhu et al., 2023). Furthermore, behavioral characteristics of exercise, such as timing and frequency, may shape its protective effects; for example, more frequent evening exercise is associated with lower levels of mobile phone addiction (Su et al., 2024).

Emerging evidence from network and longitudinal analyses further indicates that self-control functions as a key bridging variable in the relationship between exercise and mobile phone addiction, while emotional regulation and psychological resilience may also play important roles (He et al., 2025; Jin et al., 2023; Lai et al., 2025). Experimental studies reinforce these associations: basketball training and tai chi interventions have led to significant reductions in internet and smartphone addiction symptoms, alongside improvements in psychological well-being (Li et al., 2011; Yang and Zeng, 2017). Systematic reviews and meta-analyses also support the effectiveness of physical exercise interventions for reducing technology-related addictions among university students, including Chinese samples, despite heterogeneity in effect sizes (Wang Y. et al., 2025; Wang Z. et al., 2025; Yan et al., 2025). Cross-cultural research further suggests that the cognitive and emotional mechanisms underlying this relationship are consistent across contexts, indicating their potential universality (Tao et al., 2024).

Taken together, the existing literature provides converging evidence that regular physical exercise serves as a protective factor against mobile phone addiction, both directly and indirectly through cognitive, emotional, and self-regulatory pathways. In China, where smartphone penetration among college students is nearly universal, understanding this relationship has significant implications for promoting healthy behavioral patterns and designing effective intervention strategies. Based on the above evidence, this study proposes the following hypothesis:

*Hypothesis 1*: Regular physical exercise is negatively associated with mobile phone addiction tendency among Chinese college students.

### 2.2 The mediating role of trait boredom

Boredom is a negative psychological state characterized by a desire for engagement coupled with a perceived inability to find satisfying activities. This aversive experience typically stems from two psychological mechanisms: limited internal motivation and underutilized cognitive resources. Boredom is classified into state boredom and trait boredom. Trait boredom refers to a stable predisposition to frequently experience state boredom due to a chronic lack of self-initiated activity (Zhang et al., 2020). As a personality trait, trait boredom is influenced by individuals’ cognitive styles and reflects a recurring emotional response pattern.

According to the Attention Theory of Boredom, individuals with high levels of trait boredom tend to have difficulty sustaining attention and frequently experience attentional disengagement, leading to emotional discomfort and the search for substitute activities (Harris, 2000). However, these substitute behaviors often fail to engage cognitive resources meaningfully, thereby perpetuating the boredom experience (Eastwood et al., 2012).

Studies suggest that regular physical exercise is associated with lower levels of boredom (McCurdy et al., 2022). On one hand, exercise enhances energy, attention, and a sense of self-worth, thereby reducing the likelihood of experiencing trait boredom (Hüttermann and Memmert, 2012). On the other hand, regular physical exercise also positively influences individuals’ perceptions of themselves and the world (Ma et al., 2022), supporting more adaptive cognitive appraisals. Individuals who engage in regular physical exercise tend to maintain better attentional control, emotional regulation, and behavioral self-regulation (Liu et al., 2022), contributing to lower trait boredom levels (Hunter and Eastwood, 2018).

Based on the Cognitive-Emotion-Behavior Model, behavior is influenced jointly by cognitive and emotional processes (Izard, 1978). Positive cognitive appraisal facilitates positive emotional states, which in turn promote adaptive behavioral responses (Lammers, 2006). Boredom can be seen as a maladaptive emotional reaction to negative cognitive evaluations of one’s environment, while mobile phone addiction is often a behavioral response aimed at coping with such emotional discomfort (Park and Lee, 2011; Van Deursen et al., 2015). Therefore, trait boredom serves as a critical psychological antecedent of mobile phone addiction (Tong et al., 2019). Individuals with high trait boredom are more likely to engage in habitual and avoidant behaviors, such as compulsive phone use, often unconsciously investing excessive time in mobile phone activities (Zhang et al., 2019). Additionally, trait boredom has been found to mediate the relationship between various negative psychological factors (e.g., cognitive failure, poor self-regulation, and procrastination) and mobile phone addiction tendency (Elhai et al., 2018). Thus, individuals who regularly participate in physical exercise are less likely to experience boredom and, consequently, are at lower risk of developing mobile phone addiction tendency. Based on the above theoretical and empirical research results, the following hypothesis is proposed:

*Hypothesis 2*: Trait boredom mediates the relationship between regular physical exercise and mobile phone addiction tendency.

### 2.3 The moderating role of self-control

Self-control, as conceptualized by De Ridder et al. (2012), is the purposeful modulation of one’s actions to align with long-term goals while resisting short-term temptations. As a core component of the self-regulation system, self-control is considered a critical protective factor in promoting psychological and social adjustment (Pace et al., 2018; Wang et al., 2018). According to Dual-system Theory, behavior is influenced by both impulsive and inhibitory systems (Hochman, 2024). Self-control, functioning within the inhibitory system, plays a key role in regulating impulsive responses and may moderate the relationship between trait boredom and mobile phone addiction tendency.

Theoretical support for this moderating role comes from the Resilience Theory and Risk-Buffering Model, which suggest that the presence of risk factors (e.g., trait boredom) does not inevitably lead to maladaptive behaviors if individuals possess sufficient protective factors such as self-control (Fergus and Zimmerman, 2005). High levels of self-control facilitate positive cognitive and emotional responses, suppress maladaptive psychological tendency, and allow individuals to maintain attentional focus or shift it as needed (Tangney et al., 2004). Therefore, even when experiencing trait boredom, individuals with high self-control are better equipped to respond adaptively and avoid excessive phone use.

In contrast, the Self-regulation Failure Theory suggests that maladaptive behaviors in response to psychological risks often stem from an individual’s inability to effectively manage internal emotional states and external actions (Struk et al., 2016). Individuals with low self-control are more prone to problematic smartphone use when experiencing boredom. Conversely, those with high self-control are better equipped to manage potential risks and implement timely behavioral adjustments (Guo et al., 2022), thereby reducing the likelihood of developing mobile phone addiction.

Empirical evidence confirms that self-control negatively predicts various negative emotions and maladaptive behaviors, including boredom, depression, anxiety, alcohol abuse, internet addiction, and smartphone addiction (Mugon et al., 2020). Moreover, self-control can buffer the impact of negative psychological factors on individuals’ behavioral outcomes.

According to the Strength Model of Self-control, individuals who engage in regular physical exercise tend to have higher self-control capacity. Numerous empirical studies have demonstrated a positive association between regular physical exercise and self-control (Ahn and Kim, 2022; Kopp et al., 2019). For example, a 20-week program involving 8 min of daily exercise improved self-control among young adults, as measured by the Stroop test (Ludyga et al., 2018); similar improvements were observed among older adults after 12 weeks of aerobic training (Predovan et al., 2012). Acute bouts of moderate-intensity exercise involving cognitive engagement have also been shown to enhance self-control capacity (Chang et al., 2012).

Thus, regular physical exercise may strengthen college students’ self-control, enabling them to better regulate trait boredom and associated emotional responses. Students with higher self-control are more likely to adjust their emotional states and behavioral patterns when experiencing boredom, thereby reducing the likelihood of developing mobile phone addiction. Based on the above evidence, the following hypothesis is proposed:

*Hypothesis 3*: Self-control moderates the mediating effect of trait boredom in the relationship between regular physical exercise and mobile phone addiction tendency.

**Figure 1:**
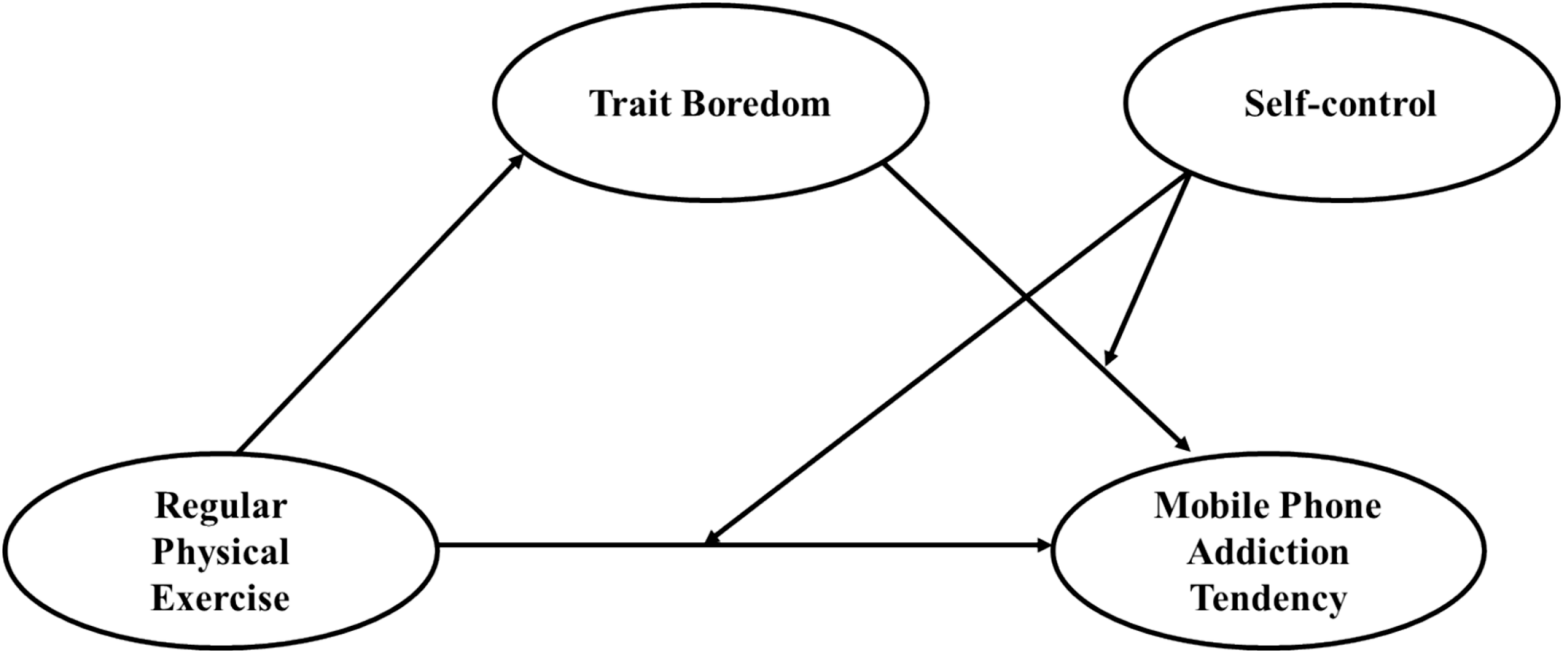
A theoretical model of regular physical exercise affects mobile phone addiction tendency.

The theoretical model constructed in this study illustrating the impact of regular physical exercise on mobile phone addiction tendency is shown in Figure 1.

## 3 Methods

### 3.1 Participants and procedures

This study was approved by the Ethics Committee of Shanghai University (ECSHU: 2025–019). Participants were recruited through convenience sampling from three universities in Shanghai: Shanghai University, Shanghai Jiao Tong University, and ShanghaiTech University. All participants provided informed consent prior to participation. Members of the research team were undertaking physical education teaching internships at these universities, where they were responsible for general public physical education courses. This setting offered convenient access to a large pool of potential participants and facilitated data collection. The rationale for selecting college students as the target population is twofold: (1) Compared to other age groups, college students generally have more discretionary time and are among the most frequent mobile phone users, making them particularly susceptible to mobile phone addiction. They also have greater opportunities to engage in regular physical exercise, increasing the likelihood of identifying individuals with consistent exercise behavior. (2) As a key group in the national talent reserve, college students require support to enhance their overall competence and avoid negative tendency such as trait boredom and mobile phone addiction, while strengthening their self-control abilities.

The survey was administered during participants’ physical education classes by trained instructors, who distributed the questionnaires via the online platform “Wenjuanxing” from April 1st to 30th, 2025. Prior to completing the survey, participants were provided with a written introduction outlining the study objectives, response instructions, privacy protection, and data usage and storage protocols. Informed consent was obtained electronically from all participants, who voluntarily participated and were free to withdraw at any time. A total of 612 responses were collected. After excluding invalid responses, such as, those completed in less than 100 s and those with identical responses on five or more consecutive items (Curran, 2016), 560 valid questionnaires were retained, yielding an effective response rate of 91.5%. Among the respondents, 47.8% were female and 54.2% were male. In the survey sample, 61.2% of participants were undergraduate students, while 38.8% were graduate students. Additionally, 33.6% majored in natural sciences, whereas 66.4% were from the humanities and social sciences.

### 3.2 Measurement tools

#### 3.2.1 Regular physical exercise

This study employed the Physical Activity Rating Scale (PARS-3) revised for Chinese college students by Liang (1994). The scale consists of three items that assess exercise intensity, duration per session, and exercise frequency. Since the original scale does not address exercise persistence, this study additionally incorporated an item from the Exercise Stage Scale developed by Marcus et al. (1992): “How long have you been regularly participating in physical exercise?” This item was used to represent the persistence of regular physical activity. In total, four items were used to comprehensively evaluate the participants’ regular physical exercise behaviors. Each item is scored on a five-point Likert scale ranging from 1 (strongly disagree) to 5 (strongly agree), with higher total scores indicating a higher level of regular physical exercise.

#### 3.2.2 Mobile phone addiction tendency

Mobile phone addiction tendency was measured using the College Students’ Mobile Phone Addiction Tendency Scale developed by Su et al. (2014), which is tailored to the Chinese university context. The scale includes 22 items, such as “Mobile phone use dominates my thoughts and behaviors” and “My excessive mobile phone use lowers my work or academic efficiency.” All items are positively worded and rated on a five-point Likert scale ranging from 1 (strongly disagree) to 5 (strongly agree). Higher scores indicate stronger mobile phone addiction tendency. Given that the scale was developed for Chinese college students, all 22 items were included in the present study.

#### 3.2.3 Trait boredom

Trait boredom was measured using the Trait Boredom Scale developed by Struk et al. (2017), and adapted for Chinese college students by Peng et al. (2019), which has demonstrated good reliability and validity. The scale comprises of 8 items, all of which were employed in the present study. Example items include “I find it hard to amuse myself,” “I need more stimulation than most people to get going,” and “I often just sit around doing nothing.” Participants responded on a 5-point Likert scale ranging from 1 (strongly disagree) to 5 (strongly agree), with higher scores indicating higher levels of trait boredom.

#### 3.2.4 Self-control

Self-control was measured using the Chinese version of the Brief Self-control Scale revised by Luo et al. (2021), which includes 7 items covering two dimensions: self-discipline and impulse control. Sample items include “I follow rules and norms” and “I can stop myself from acting on impulses.” Items are rated on a five-point Likert scale ranging from 1 (strongly disagree) to 5 (strongly agree). Items 2, 4, 6, and 7 are reverse-scored. After transformation, higher scores indicate greater levels of self-control. As the scale has demonstrated good reliability and validity among Chinese college students, all seven original items were used without modification in this study.

### 3.3 Reliability and validity

The Cronbach’s *α* coefficients for all scales exceeded 0.70, indicating that the measurement instruments demonstrated acceptable internal consistency and good reliability across all variables (Table 1).

**Table 1:** Reliability analysis of all variables.

Variable	Cronbach’s α
Regular physical exercise	0.735
Trait boredom	0.894
Mobile phone addiction tendency	0.931
Self-control	0.830

Confirmatory factor analysis indicated that the measurement model comprising regular physical exercise, trait boredom, self-control, and mobile phone addiction tendency exhibited a good model fit: χ^2^/*df* = 2.389, RMSEA = 0.053, CFI = 0.946, IFI = 0.948, TLI = 0.942. These results demonstrate that the measurement tools possess satisfactory structural validity.

### 3.4 Data analysis

The statistical analysis was conducted using SPSS 26.0. To ensure the quality of measurement, preliminary tests of reliability and construct validity were performed for the primary instruments: regular physical exercise, trait boredom, self-control, and mobile phone addiction tendency. Cronbach’s α coefficients were calculated to assess internal consistency reliability, while confirmatory factor analysis was used to evaluate structural validity. Descriptive statistics and Pearson correlation analysis were conducted to examine variable distributions and the initial relationships among the variables.

To formally test the research hypotheses, hierarchical regression analysis was conducted to assess the predictive effect of regular physical exercise on mobile phone addiction tendency. The mediating role of trait boredom and the moderating role of self-control were further examined using PROCESS macro. Bootstrap procedures with 5,000 resamples and 95% confidence intervals (CI) were employed to estimate indirect and moderated effects, ensuring the robustness of the mediation and moderation tests.

## 4 Results

### 4.1 Common method bias test

To assess the potential for common method bias, Harman’s single-factor test was conducted. The results revealed that six factors had eigenvalues greater than 1, and the first factor accounted for 29.863% of the total variance, which is below the critical threshold of 40%. This indicates that common method bias was not significant in this study.

### 4.2 Descriptive statistics and correlation analysis

Pearson correlation analysis indicated several significant associations among the main study variables (Table 2). Regular physical exercise was negatively correlated with both mobile phone addiction tendency (*r* = −0.209, *p* < 0.01) and trait boredom (*r* = −0.273, *p* < 0.01), while positively correlated with self-control (*r* = 0.566, *p* < 0.01). Trait boredom was negatively correlated with self-control (*r* = −0.365, *p* < 0.01) and positively correlated with mobile phone addiction tendency (*r* = 0.549, *p* < 0.01). In addition, self-control was negatively correlated with mobile phone addiction tendency (*r* = −0.297, *p* < 0.01).

**Table 2:** Descriptive statistics and correlation analysis.

Variable	Mean	SD	1	2	3	4
1. Regular physical exercise	2.8768	0.96	1			
2. Mobile phone addiction tendency	3.0991	0.95	−0.209^**^	1		
3. Trait boredom	2.9421	0.96	−0.273^**^	0.549^**^	1	
4. Self-control	2.9964	0.75	0.566^**^	−0.297^**^	−0.365^**^	1

**p < 0.01.

### 4.3 Tests of research hypothesis

#### 4.3.1 Test of the mediating effect

This study employed Hayes’ SPSS PROCESS macro (Model 4) to examine the mediating role of trait boredom in the association between regular physical exercise and mobile phone addiction tendency. Prior to the regression analyses, control variables were coded as follows: gender (0 = female, 1 = male) was directly entered as a binary variable, while grade level and academic major, as categorical variables with more than two categories, were dummy coded before being entered into the model. The results indicated that regular physical exercise was negatively associated with trait boredom (*β* = −0.301, *t* = −5.460, *p* < 0.001, 95% CI [−0.410, −0.193]), and trait boredom was positively associated with mobile phone addiction tendency (*β* = 0.500, *t* = 15.269, *p* < 0.001, 95% CI [0.436, 0.565]). Even after trait boredom was included, regular physical exercise retained a negative association with mobile phone addiction tendency (*β* = −0.192, *t* = −4.386, *p* < 0.001, 95% CI [−0.278, −0.106]; Table 3).

**Table 3:** Mediating effect of trait boredom on the relationship between regular physical exercise and mobile phone addiction tendency.

Dependent variable	Predictor variable	*R*	*R* ^2^	*F*	*β*	LLCI	ULCI	*t*
Trait Boredom	Gender				−0.213	−0.429	0.002	−1.950
	Grade				−0.022	−0.063	0.019	−1.066
	Major				0.169	−0.036	0.374	1.621
	Regular physical exercise				−0.301	−0.410	−0.193	−5.460^***^
		0.301	0.091	8.782^***^				
Mobile phone addiction tendency	Gender				0.006	−0.127	0.139	0.086
	Grade				0.029	0.004	0.054	2.255
	Major				0.087	−0.040	0.213	1.346
	Regular Physical Exercise				−0.041	−0.110	−0.019	−1.859^**^
	Trait Boredom				0.500	0.436	0.565	15.269^***^
		0.660	0.435	54.090^***^				
Mobile phone addiction tendency	Gender				−0.101	−0.271	0.069	−1.165
	Grade				0.018	−0.015	0.050	1.080
	Major				0.171	0.009	0.333	2.073
	Regular physical exercise				−0.192	−0.278	−0.106	−4.386^***^
		0.245	0.060	5.622^***^				

^***^p < 0.001, ^**^p < 0.01.

Further analysis of the indirect effect revealed that the mediating effect of trait boredom was significant (indirect effect = −0.151, *p* < 0.001), with a 95% CI of [−0.215, −0.092]. The indirect effect accounted for 78.65% of the total effect (Table 4), indicating that trait boredom plays a partial mediating role. In other words, regular physical exercise not only directly reduces mobile phone addiction tendency but also exerts an indirect effect by alleviating individuals’ trait boredom.

**Table 4:** Effect size of the mediating role of trait boredom.

Path	Effect size	Boot SE	LLCI	ULCI	Proportion
Indirect effect	−0.151	0.032	−0.215	−0.092	78.65%
Direct effect	−0.041	0.041	−0.1208	−0.019	21.35%
Total effect	−0.192	0.241	2.980	3.930	

#### 4.3.2 Test of the moderating effect

Multiple regression analysis was conducted to examine the moderating effect of self-control on the relationship between trait boredom and mobile phone addiction tendency, as well as between regular physical exercise and mobile phone addiction tendency. After controlling for gender, grade level, and academic major, the regression results showed that trait boredom was positively associated with mobile phone addiction tendency (*β* = 0.473, *t* = 13.722, *p* < 0.001), while self-control was negatively associated with mobile phone addiction tendency (*β* = −0.123, *t* = −2.131, *p* < 0.05). These results suggest that individuals reporting higher trait boredom also tended to report stronger mobile phone addiction tendency, while those with higher self-control tended to report weaker addiction tendency.

Further analysis revealed a significant interaction effect between trait boredom and self-control (*β* = −0.116, *t* = −2.670, *p* < 0.01), indicating that self-control moderated the relationship between trait boredom and mobile phone addiction tendency. Specifically, the positive association between trait boredom and mobile phone addiction tendency was attenuated at higher levels of self-control. In contrast, the interaction between regular physical exercise and self-control was not significant (*β* = −0.021, *t* = −0.366, *p* > 0.05), indicating that self-control did not significantly moderate the direct association between regular physical exercise and mobile phone addiction tendency (Table 5).

**Table 5:** Moderating effect of self-control on the relationship between trait boredom and mobile phone addiction tendency.

Dependent variable	Predictor variable	*R*	*R* ^2^	*F*	*β*	LLCI	ULCI	*t*
Mobile phone addiction tendency	Gender				−0.018	−0.149	0.114	−0.266
	Grade				−0.135	−0.258	−0.013	−2.166^*^
	Major				−0.097	−0.222	0.028	−1.533
	Regular physical exercise				−0. 344	−0.511	−0.026	−1.211^***^
	Trait boredom				0.473	0.405	0.541	13.722^***^
	Self-control				−0.123	−0.236	−0.009	−2.131^*^
	Regular physical exercise × Self-control				−0.021	−0.130	0.089	−0.366
	Trait boredom × Self-control				−0.116	−0.031	−0.202	−2.670^**^
		0.675	0.455	36.374				

^***^p < 0.001, ^**^p < 0.01, ^*^p < 0.05.

**Figure 2:**
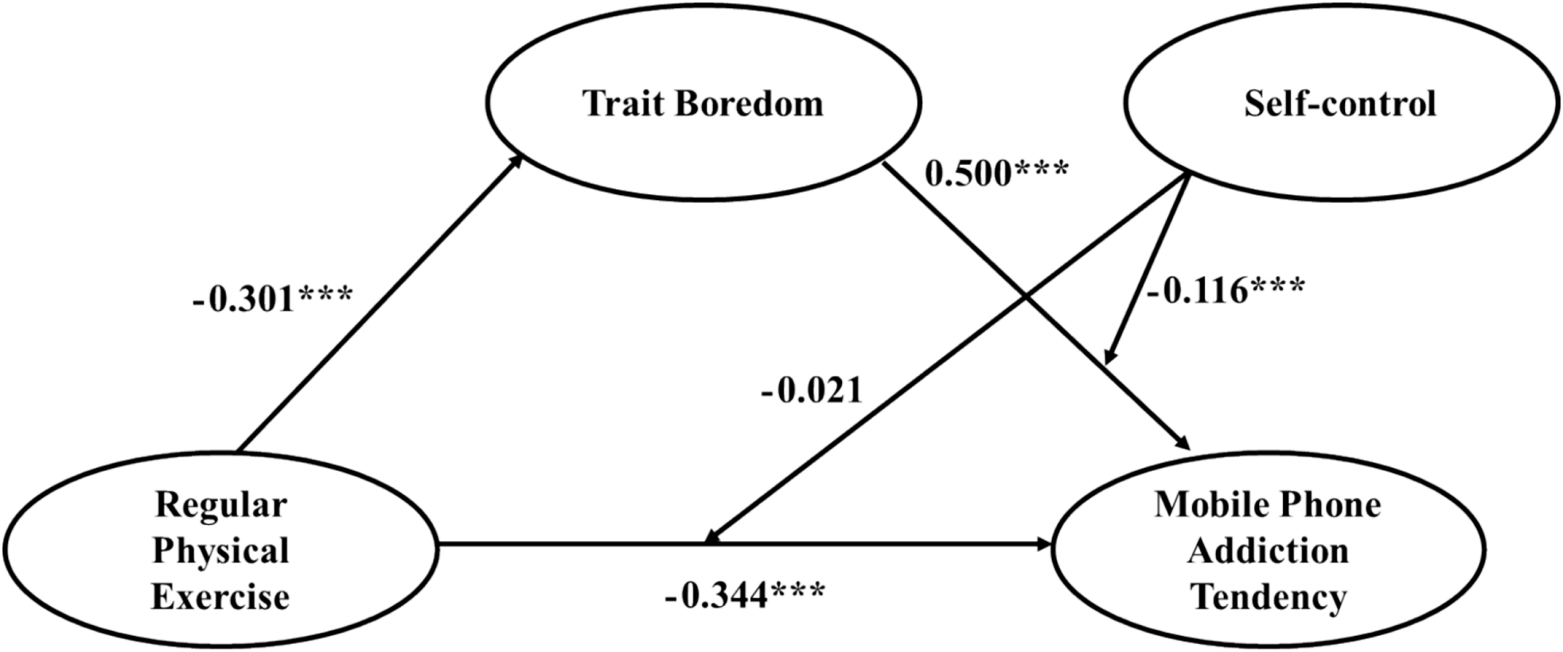
Role of trait boredom and self-control in the relationship between regular physical exercise and mobile phone addiction tendency. ***p<0.001.

In summary, the findings are consistent with the interpretation that self-control serves as a protective factor: it is directly related to lower mobile phone addiction tendency and also buffers the strength of the association between trait boredom and addictive behaviors (Figure 2).

## 5 Discussion

### 5.1 The relationship between regular physical exercise and mobile phone addiction tendency

The present study found a significant negative association between regular physical exercise and mobile phone addiction tendency among college students, indicating that students who engage more consistently in physical exercise are less likely to exhibit addictive patterns of mobile phone use. This finding is consistent with previous research (Li et al., 2020). Existing study has demonstrated that regular physical exercise plays a crucial role in mitigating addictive behaviors, including internet and smartphone addiction (Zhong et al., 2021). This effect may be attributed to two primary mechanisms. First, regular physical exercise consumes a portion of individuals’ discretionary time, thereby reducing the time available for mobile phone use and potentially lowering the risk of mobile phone addiction. Second, regular physical exercise has been shown to activate the brain’s reward system and regulate the stress response, which enhances positive affect and mitigates psychological distress. Together, these psychophysiological benefits contribute to improved overall well-being (Wunsch et al., 2017).

Given that online gaming has been identified as one of the core drivers of mobile phone addiction among college students (Abbasi et al., 2021), regular physical exercise may serve as an effective behavioral substitute. By satisfying students’ psychological needs for emotional regulation and pleasurable experiences, regular physical exercise may offer a functional alternative to mobile phone use and help reduce the likelihood of addiction.

### 5.2 The mediating role of trait boredom

This study confirmed that trait boredom significantly and positively affects mobile phone addiction tendency, a finding consistent with previous research (Wegmann et al., 2018). Prior studies have shown that boredom is often accompanied by impaired attentional control and emotional fluctuation, prompting individuals to engage in frequent mobile phone use in pursuit of immediate gratification. Over time, this pattern may evolve into compulsive or dependent behavior (Eastwood et al., 2007; Smallwood et al., 2009).

Furthermore, the results indicated that trait boredom mediates the relationship between regular physical exercise and mobile phone addiction tendency among college students. Specifically, individuals who regularly engage in physical exercise tend to report lower levels of trait boredom, which in turn is associated with a reduced mobile phone addiction tendency. This finding aligns with previous research suggesting that trait boredom may serve as a psychological bridge linking social adaptability and addictive behaviors (Yang et al., 2021).

From the perspective of Cognitive-Behavioral Theory, this mediation effect can be further explained. According to the Cognitive–Emotion–Behavior Model, emotional experiences and behavioral responses are not direct reactions to external events but are shaped by individuals’ cognitive appraisals of those events (Paykel, 1987). A more positive cognitive style helps buffer negative emotion such as boredom, anxiety, and depression, thereby enhancing individuals’ emotional regulation abilities (Pan et al., 2022). Similarly, the Attention Theory of Boredom posits that difficulty in maintaining attention and a tendency toward attentional disengagement are central mechanisms underlying boredom experiences. When these cognitive patterns persist, they may solidify into stable personality traits over time (Eastwood et al., 2012; Harris, 2000).

Notably, physical exercise appears to exert a positive regulatory effect along this pathway. Previous study has suggested that regular physical exercise enhances individuals’ attentional control and cognitive flexibility and reduces the accumulation of negative emotions (Hüttermann and Memmert, 2012). The present study indicates that regular physical exercise may be linked to more efficient cognitive processing of external stimuli, potentially alleviating boredom and decreasing compensatory mobile phone use. In this way, trait boredom functions as a key mediating variable through which physical exercise influences mobile phone addiction tendency.

These findings not only deepen the understanding of the psychological mechanisms underlying mobile phone addiction but also provide theoretical and practical guidance for campus-level interventions. By fostering an environment that encourages regular physical exercise, universities may help students improve attention and emotional regulation, thereby reducing the likelihood of maladaptive behaviors such as mobile phone addiction.

### 5.3 The moderating role of self-control

This study further revealed that self-control moderates the indirect effect of regular physical exercise on mobile phone addiction tendency via trait boredom. Specifically, self-control moderated the second stage of the mediation pathway, namely, the link between trait boredom and mobile phone addiction tendency. In other words, as college students’ self-control improves, the predictive power of trait boredom on mobile phone addiction decreases. This finding is consistent with previous studies that identify self-control as a buffer against the impact of negative psychological factors on irrational behaviors (Yanhui et al., 2017). Moreover, the moderating effect of self-control was more pronounced among individuals with higher levels of trait boredom.

These findings provide empirical support for and further enrich the theoretical implications of Resilience Theory and the Risk Buffering Model, both of which posit that self-control can function as a protective factor that mitigates the adverse effects of risk factors (e.g., trait boredom) on emotional dysregulation and maladaptive behaviors (Ellis, 2019). According to the Theory of Self-regulatory Failure, irrational behaviors often arise from individuals’ inability to effectively regulate their emotions and behaviors in the face of risk or stress (De Ridder et al., 2012). Individuals with high self-control are more capable of evaluating and adjusting their emotional and behavioral states in a timely manner and can maintain focused attention for extended periods (Mugon et al., 2020). Such regulatory capacity helps reduce the likelihood of developing mobile phone addiction. Conversely, individuals with low self-control struggle to sustain attention or reallocate cognitive resources when experiencing boredom, which intensifies the effect of boredom on mobile phone addiction tendency (Sato et al., 2018).

Interestingly, the present study found that self-control did not significantly moderate the direct relationship between regular physical exercise and mobile phone addiction tendency. This suggests that the protective effect of regular physical exercise on mobile phone addiction tendency may operate independently of self-control. Prior empirical studies have shown that trait self-control positively predicts individuals’ physical exercise levels, and this relationship may be mediated by the development of stable exercise habits. Specifically, individuals with high self-control are more likely to cultivate consistent and regular exercise behaviors (Gillebaart and Adriaanse, 2017). Therefore, even among individuals with varying levels of self-control, regular physical exercise may exert a beneficial effect on reducing mobile phone addiction tendency. Another possible explanation is that individuals who engage in regular physical exercise tend to possess higher self-control by default, which may overshadow the moderating role of self-control in the physical exercise–addiction relationship.

These findings offer important implications for the prevention and intervention of mobile phone addiction among college students. Targeted efforts should be made to address trait boredom, such as promoting time management skills and attention training, to help students effectively cope with boredom and reduce unnecessary mobile phone use. In addition, self-control remains a crucial protective factor, especially for individuals with high levels of trait boredom. Therefore, universities should strengthen students’ self-monitoring and behavioral regulation abilities through mental health education and psychological skills training.

## 6 Conclusion

This study provides empirical evidence that regular physical exercise is negatively associated with mobile phone addiction tendency among Chinese college students, with trait boredom potentially serving as a mediating mechanism. Furthermore, self-control appears to function as a protective factor by moderating the indirect pathway, buffering the impact of trait boredom on mobile phone addiction tendency. However, self-control did not significantly moderate the direct association between regular physical exercise and mobile phone addiction tendency.

In Chinese universities, internet addiction has become a prevalent phenomenon, and the resulting social isolation and interpersonal fragmentation are increasingly undermining students’ prosocial behaviors. Existing research indicates that regular physical exercise can effectively enhance students’ self-control and, in turn, reduce trait boredom, thereby providing a positive intervention against mobile phone addiction. Although the present study found that self-control did not significantly moderate the direct effect of physical exercise on mobile phone addiction, the role of physical exercise in strengthening self-control has been widely recognized in the literature. As self-control improves, students are better able to cope with boredom and resist addictive behaviors. Taken together, these findings suggest that in the university context, regular physical exercise not only alleviates trait boredom but may also, through the enhancement of self-control, play an important role in preventing and reducing problematic internet and mobile phone use.

### 6.1 Implications

The results of this study highlight the practical importance of integrating regular physical exercise into university settings as a strategy to address mobile phone addiction among college students. Physical education should not be limited to promoting physical fitness but should also serve as a vehicle for fostering psychological well-being and behavioral self-regulation. By broadening educational objectives beyond skill acquisition and physical conditioning, universities can help students develop healthy lifestyle habits and stronger self-control capacities.

From an educational perspective, regular participation in physical activity can reduce boredom and enhance self-regulation, thereby supporting healthier learning and living patterns. These psychological benefits are particularly valuable in mitigating issues associated with excessive smartphone use, such as decreased attention and reduced academic efficiency.

In practical application, universities may consider institutionalizing specific strategies to encourage students to incorporate physical exercise into their daily routines. Examples include implementing “phone-free exercise periods” and organizing thematic initiatives that combine “physical activity and mental health.” Such measures can contribute to creating a supportive campus culture that not only enhances students’ physical health but also reduces the prevalence of mobile phone addiction.

### 6.2 Limitations and future directions

Grounded in the Cognitive–Emotion–Behavior model, the Resilience Theory, and the Theory of Self-regulation Failure, this study developed a moderated mediation model to investigate the mechanisms by which regular physical exercise affects mobile phone addiction tendency among college students. The results demonstrated that regular physical exercise not only directly reduces students’ mobile phone addiction tendency but also exerts an indirect effect by lowering trait boredom. Furthermore, self-control significantly moderates the link between trait boredom and mobile phone addiction tendency, enriching the theoretical framework of exercise-based psychological and behavioral regulation.

These findings suggest that universities should place greater emphasis on the psychological benefits of regular physical exercise, cultivate a campus culture that encourages regular participation in physical exercise, and integrate physical exercise promotion with psychological education and counseling services. Enhancing students’ self-control abilities through targeted interventions may further strengthen their capacity to manage cognitive, emotional, and behavioral challenges associated with mobile phone overuse. The present study also offers empirical evidence for the design of mental health promotion initiatives and healthy lifestyle programs on campus.

Despite its theoretical and practical contributions, this study has several limitations. First, the use of a cross-sectional design and self-reported data limits the ability to draw causal inferences. Future research may adopt longitudinal designs or experimental interventions to better capture the dynamic relationships among variables. Second, all measures were based on subjective self-report scales, which may be subject to social desirability bias or response tendencies. Future studies could incorporate behavioral observations, digital tracking, or third-party evaluations to enhance measurement reliability. Third, the sample was primarily drawn from universities in Eastern China, which may limit the generalizability of the findings. Future research should include more diverse geographic regions and populations to improve the external validity and applicability of the results.

## Data Availability

The raw data supporting the conclusions of this article will be made available by the authors, without undue reservation.
